# Rat prostate tumors induce DNA synthesis in remote organs

**DOI:** 10.1038/s41598-022-12131-6

**Published:** 2022-05-12

**Authors:** Sofia Halin Bergström, Marie Lundholm, Annika Nordstrand, Anders Bergh

**Affiliations:** Department of Medical Biosciences, Pathology, Umeå University, Building 6M, second floor, 901 87 Umeå, Sweden

**Keywords:** Biomarkers, Oncology, Prostate, Cancer, Cancer microenvironment, Tumour biomarkers

## Abstract

Advanced cancers induce systemic responses. However, if such systemic changes occur already when aggressive tumors are small, have not been thoroughly characterized. Here, we examined how localized prostate cancers of different sizes and metastatic potential affected DNA synthesis in the rest of the prostate and in various remote organs. Non-metastatic Dunning R-3327 G (G) tumor cells, metastatic MatLyLu (MLL) tumor cells, or vehicle were injected into the prostate of immunocompetent rats. All animals received daily injections of Bromodeoxyuridine (BrdU), to label cells/daughter cells with active DNA synthesis. Equal sized G- and MLL-tumors, similarly increased BrdU-labeling in the prostate, lymph nodes and liver compared to tumor-free controls. Prior to metastasis, MLL-tumors also increased BrdU-labeling in bone marrow and lungs compared to animals with G-tumors or controls. In animals with MLL-tumors, BrdU-labeling in prostate, lungs, brown adipose tissue and skeletal muscles increased in a tumor-size-dependent way. Furthermore, MLL-tumors induced increased signs of DNA damage (γH2AX staining) and accumulation of CD68 + macrophages in the lungs. In conclusion, small localized prostate cancers increased DNA synthesis in several remote tissues in a tumor type- and size-dependent way. This may suggest the possibility for early diagnosis of aggressive prostate cancer by examining tumor-induced effects in other tissues.

## Introduction

Prostate cancer is a common cancer with highly variable outcome. Many of the tumors are harmless and cause no symptoms—making treatment unnecessary—whereas others are lethal and need to be diagnosed and treated as early and effectively as possible. Current diagnostic methods, like serum-PSA, magnetic resonance imaging and histological examination of subsequent prostate needle biopsies, cannot always separate localized harmless tumors from those needing treatment^1,2^. Improved diagnostic methods, allowing detection of potentially lethal prostate cancers at an early stage are therefore required.

Tumor cells interact with other cells in the tumor microenvironment, like fibroblasts, vascular cells and immune cells—that in turn influence tumor growth and progression^3,4^. Consequently, several alterations in the tumor stroma have been linked to prostate cancer prognosis^4^. Prostate cancers affect, not only the nearby tumor stroma, but the whole prostate tissue^5^. Already prior to metastasis, fast-growing and highly metastatic tumors induce different and more pronounced changes in the surrounding normal prostate tissue and regional lymph nodes compared to similar sized low-metastatic tumors^6–9^. Changes commonly found in the benign parts of a tumor-bearing prostate are; increased vascular growth, extracellular matrix alterations, increased inflammation, altered gene expression profiles, and a reduced response to androgen withdrawal—with some changes found already when the tumor is less than one millimeter in diameter^6–16^. Similar changes, coupled to tumor aggressiveness and outcome, have also been found in the benign parts of the prostate and regional lymph nodes in prostate cancer patients^7,10,13,14,16–20^. We have termed this phenomenon “Tumor Instructed/Indicating Normal Tissue” or shortly TINT-changes.

Tumors also signal to remote tissues, like the liver and bone marrow, to recruit proteins, inflammatory cells and mesenchymal cells^21^. Furthermore, circulating tumor factors, for example tumor-derived microvesicles, reshape and prepare remote tissues for subsequent metastatic growth, so called pre-metastatic niche formation^22^. Together these studies suggest that tumor aggressiveness could perhaps be evaluated by examining how various adjacent and remote tissues are “tinted” by the presence of localized tumors. This could be particularly useful if changes can be detected already when potentially lethal tumors are small and curable.

The aim of this study was to examine if small intra-prostatic rat prostate tumors affect distant tissues prior to metastatic establishment. Two rat prostate tumor types, G or MLL, were implanted into the prostate of fully immunocompetent animals^6^. The G tumors have low metastatic potential with no detectable metastases even after 8–12 weeks when growing in the prostate^23^. MLL tumors have high metastatic potential to lymph nodes and lungs^24^, with the first signs of microscopically detectable metastases at day 14 in lymph nodes^8^ and at day 18 in lungs (unpublished) in our orthotopic model. MLL metastases are sometimes found in liver^25^ but have not been shown in other organs^24^.

Results from tumor-bearing animals were compared to both vehicle-injected and treatment naive tumor-free control rats. All animals received daily systemic administration of BrdU for 10 days, a time-point were no microscopic metastases have previously been detected^9^. We hypothesized that BrdU, which is incorporated in cells with active DNA synthesis and passed on to daughter cells, is a sensitive method to mark all sites in the body with tumor-induced responses in DNA repair/synthesis/cell proliferation^26^. In addition, macrophage (CD68) density and signs of DNA damage (γH2AX staining)^27^ were also analyzed in selected organs.

## Results

### Small orthotopic prostate tumors increase BrdU-labeling in the benign parts of the prostate and in distant organs

To examine if BrdU-labeling in near and remote organs was related to tumor type, we compared animals with intraprostatic G tumors to animals with intraprostatic MLL tumors (referred to as G-small and MLL-small, n = 7 in each group). To examine if BrdU-labeling also was related to tumor size, we compared animals with MLL-small tumors to animals with larger intraprostatic MLL tumors (MLL-large, n = 7). All tumor bearing animals were also compared to tumor-free control animals (n = 6) that were given an intraprostatic injection of vehicle (RPMI).

Ten days after tumor cell injection, the G-small and the MLL-small tumors had reached similar sizes. The tumor cross sectional area was 2.8 ± 1.0 mm^2^ of G-small tumors and 3.0 ± 0.9 mm^2^ of MLL-small tumors (mean ± SEM, p = 0.645), with G-small tumors occupying in average 18 ± 6% and MLL-small tumors 18 ± 7% (mean ± SEM) of the right ventral prostate lobe volume. The MLL-large tumors were still organ confined but were significantly larger than the MLL-small tumors (12.1 ± 3.1 mm^2^ vs. 3.0 ± 0.9 mm^2^, p = 0.006, mean ± SEM). Consequently, all tumors were small and surrounded by non-malignant normal prostate tissue and no signs of metastases could be detected by microscopic examination in any organ examined. However, the presence of very low numbers of single disseminated tumor cells in remote organs at this time-point could not be fully excluded.

As anticipated, numerous cells were BrdU-labeled in tissues with high cell turn-over such as tumors, lymph nodes, and the bone marrow, but labeled cells could also be identified and quantified in more stationary tissues such as the prostate, liver and lungs (Fig. S1).

**Figure 1:**
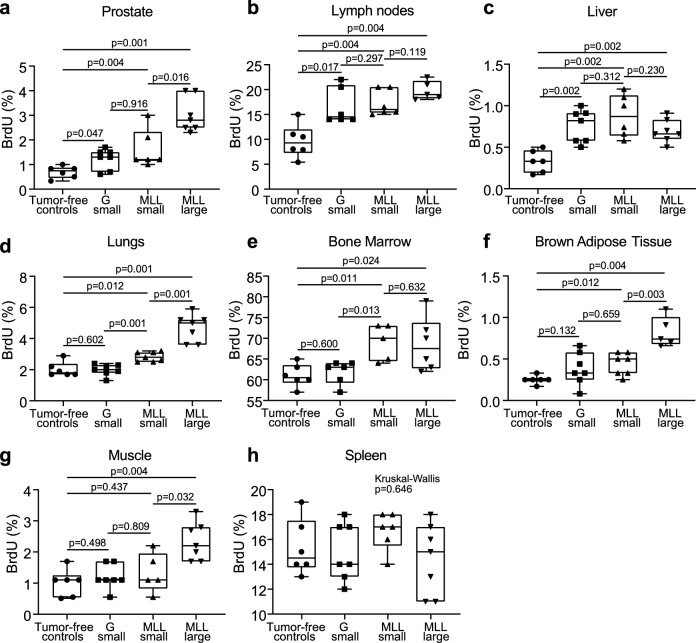
BrdU-labeling in various organs in animals with intraprostatic tumors. Box plots showing the volume density (%) of BrdU-labeled cells in the ventral prostate (**a**), regional lymph nodes (**b**), liver (**c**), lungs (**d**), bone marrow (**e**), brown adipose tissue (**f**), skeletal muscle (**g**), and spleen (**h**) in vehicle-injected tumor-free control rats and in rats with intra-prostatic G-small, MLL-small, or MLL-large tumors. Non-metastatic G-small tumors significantly increased BrdU-labeling in the prostate, lymph nodes, and liver. Metastatic MLL tumors significantly increased BrdU-labeling in the prostate, lymph nodes, liver, bone marrow, lungs, and brown adipose tissue. BrdU-labeling was significantly increased in a tumor-size dependent way in the prostate, lungs, brown adipose tissue and skeletal muscle but not in lymph nodes, liver, bone marrow or spleen. For each tissue examined, all four groups were compared using the non-parametric Kruskal–Wallis H test. If the multiple comparison showed significance (p < 0.05), the non-parametric Mann–Whitney U test was used to compare two groups.

Animals with intra-prostatic G or MLL tumors had increased BrdU-labeling in nearby tissues such as the surrounding benign prostate tissue and draining lymph nodes compared to tumor-free controls (Fig. 1a,b, Fig. S1). BrdU-labeling index was also significantly higher in the prostate but not in the regional lymph nodes, in animals with MLL-large tumors compared to animals with MLL-small tumors (Fig. 1a,b, Fig. S1).

BrdU-labeling was also increased in the liver of tumor bearing animals compared to tumor-free controls (Fig. 1c, Fig. S1). However, BrdU-labeling in the liver did not significantly increase with increased MLL tumor size (Fig. 1c, Fig. S1). In animals with MLL tumors, BrdU-labeling was also increased in the lungs and bone marrow compared to animals with G tumors or tumor-free controls (Fig. 1d,e, Fig. S1). BrdU-labeling index was significantly higher in the lungs but not in the bone marrow, in animals with MLL-large tumors compared to animals with MLL-small tumors (Fig. 1d,e, Fig. S1). Increased BrdU in brown adipose tissue was also found in animals with MLL tumors compared to controls, and BrdU-labeling increased significantly with increased MLL-tumor size (Fig. 1f, Fig. S1). Muscle tissue did not show any changes in BrdU-labeling in animals with G-small or MLL-small tumors compared to tumor-free controls but increased significantly with increased MLL tumor size (Fig. 1g, Fig. S1). BrdU-labeling was unaffected in the spleen (Fig. 1h).

Taken together, this suggests that tumors, already when small, induce DNA synthesis in nearby tissues but also distantly in the liver, regardless of tumor metastatic potential. Small organ-confined tumors with metastatic potential also induce DNA synthesis in distant organs such as lungs, bone marrow, and brown fat prior to metastatic establishment.

We then examined if surgery and intra-prostatic injection of vehicle could affect BrdU-labeling in the control rats by comparing them to fully treatment-naïve rats. A slight increase in BrdU-labeling was observed in the prostate and in the spleen (Table S1). In all other organs examined, the BrdU-labeling was not significantly altered between in vehicle-injected and treatment naïve animals (Table S1).

All animals that had undergone surgery (vehicle or tumor cell injection) had lost weight at day 10 compared to day 0, which was not seen in treatment naïve animals (relative weight at day 10 to day 0, mean ± SEM: Naïve; 0.98 ± 0.01, Vehicle; 0.95 ± 0.01, G-tumors; 0.92 ± 0.01, small MLL-tumors; 0.93 ± 0.01, large MLL-tumors; 0.93 ± 0.01), showing that handling and surgery per se resulted in animal weight reduction. However, animal weight reduction was not significantly different in any of the groups with surgery (p = 0.145, Kruskal–Wallis test), showing that tumor growth had not significantly lowered the body weight at the end of the study, and the animals were not cachexic.

### Increased BrdU-labeling in various cell types in benign prostate, liver and lungs

**Figure 2:**
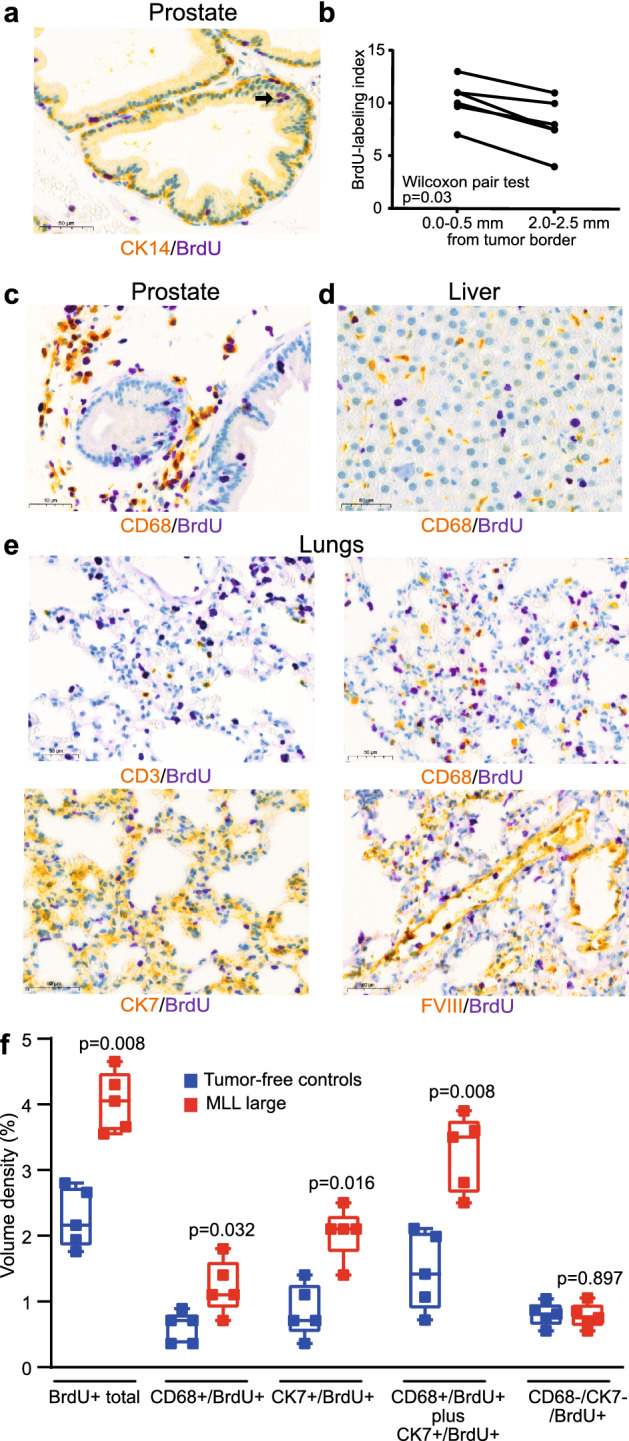
BrdU-labeling in various cell types in prostate, liver and lungs. (**a**) Sections from benign prostate glands in a rat with an intraprostatic MLL-tumor double-stained for BrdU (purple nucleus) and basal epithelial cell (CK14, dark yellow cytoplasm). Some basal CK14 + epithelial cells and some luminal epithelial cells (arrow) were BrdU +. (**b**) Graph showing the fraction (%) of BrdU-labeled benign prostate epithelial cells close to the MLL-tumor border (0–0.5 mm) or further way from the tumor border (2.0–2.5 mm). Prostate glands close to the tumor border had a significantly higher fraction of BrdU-labeling than more remote glands (Wilcoxon paired test). **c**) Prostate tissue double-stained for BrdU (purple nucleus) and CD68 (yellow cytoplasm). Some infiltrating CD68 + cells were BrdU +. (**d**) Liver tissue double-stained for BrdU (purple nucleus) and CD68 (yellow cytoplasm) in an animal with an intra-prostatic MLL tumor. Most BrdU + cells appeared to be hepatocytes whereas CD68 + cells were seldom BrdU-labeled. (**e**) Lung tissue immune-stained for; (1) BrdU (purple nucleus) and CD3 (yellow cytoplasm), 2) BrdU (purple nucleus) and CD68 (yellow cytoplasm), (3) BrdU (purple nucleus) and CK7 (yellow cytoplasm), and (4) BrdU (purple nucleus) and factor VIII (yellow cytoplasm) in an animal with an intra-prostatic MLL tumor. Several different cell types in the lung including pneumocytes (CK7 +) were BrdU + . (**f**) Box plots showing volume densities (%) of total BrdU-labelled cells, BrdU + macrophages (CD68 + /BrdU +), BrdU + pneumocytes (CK7 + /BrdU +), the sum of BrdU + macrophages and BrdU + pneumocytes (CD68 + /BrdU + plus CK7 + /BrdU +), and other BrdU + cells (CD68-/CK7-/BrdU +) in lungs of animals with MLL-large tumors or in tumor-free controls (Mann–Whitney U test).

We then explored in what cell types BrdU-labeling occurred. In the benign prostate tissue, some basal (CK14 +) and luminal (CK18 +) prostate epithelial cells were BrdU +, suggesting that tumors induce remodeling of the glandular epithelium (Fig. 2a). Epithelial BrdU-labeling was slightly but significantly higher in the normal prostate tissue nearby MLL-tumors compared to prostate tissue further away (Fig. 2b), demonstrating a gradient response. In line with our previous study^6^, we also observed BrdU + endothelial and mural vascular cells, especially in animals with MLL-tumors (data not shown). As previously shown^6^, animals with MLL-tumors also had markedly increased numbers of macrophages infiltrating the normal parts of their prostates. Some of the CD68 + macrophages were BrdU + whereas others where BrdU- (Fig. 2c).

An evenly distributed (non-focal) BrdU-labeling was observed in both liver and lungs. In the liver, BrdU + cells were mainly hepatocytes while CD68 + Kupffer cells were generally BrdU negative (Fig. 2d).

In the lungs, double staining with BrdU and; (1) CD3 + T-lymphocytes, (2) CD68 + macrophages, (3) Cytokeratin 7 + (CK7) pneumocytes, and (4) factor VIII + endothelium, showed BrdU-labeling in several cell types (Fig. 2e). In MLL-tumors, all tumor cells were CK7- and CD68-, while almost all tumor cells were BrdU + (data not shown). Collectively, tumors appear to induce DNA synthesis in various different cell types in the lung, but most prominent in pneumocytes.

We then quantified the total volume density of BrdU + cells, and the volume densities of CK7 + /BrdU + and CD68 + /BrdU + cells in selected lungs from rats with orthotopic MLL-large tumors or from tumor-free controls. Again, volume density of total BrdU + cells in the lungs was significantly higher in rats with MLL tumors compared to controls (Fig. 2f). In addition, volume densities of BrdU + macrophages (CD68 + /BrdU +) and BrdU + pneumocytes (CK7 + /BrdU +) were also significantly higher in animals with orthotopic MLL-tumors compared to tumor-free controls (Fig. 2f). BrdU + macrophages and BrdU + pneumocytes together constituted approximately 80% of the BrdU + cells in lungs of animals with MLL-tumors, and were, when added together, also significantly higher in animals with MLL-tumors compared to controls (Fig. 2f). The volume density of remaining BrdU + cells (CD68-/CK7-/BrdU +) was low and did not significantly differ between animals with MLL-tumors and tumor-free controls (Fig. 2f). This suggests that the increased BrdU-labelling in the lungs in animals with MLL-tumors mainly occurs in pneumocytes and macrophages, and if single BrdU + MLL tumor cells are present in the lungs their numbers must be very low.

### Macrophages and DNA damage in liver and lungs

To analyze if increased BrdU-labeling in the liver and lungs was coupled to increased inflammation and/or DNA damage, we quantified the densities of macrophages (CD68) and γH2AX positive cells.

**Figure 3:**
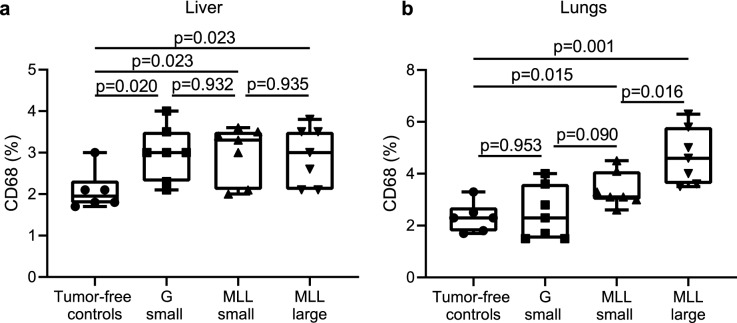
Macrophage densities in liver and lungs in animals with intraprostatic tumors. Box plots showing the volume density (%) of CD68 + cells in livers (**a**) and lungs (**b**) in animals with intraprostatic G- or MLL-tumors. The density of CD68 + cells was significantly increased in lungs by MLL-tumors compared to controls and in a tumor-size dependent way. The non-parametric Kruskal–Wallis H test was used for multiple comparison, if significant (p < 0.05), the non-parametric Mann–Whitney U test was used to compare two groups.

Animals with both types of intra-prostatic tumors had increased density of CD68 + macrophages in the liver compared to controls (Fig. 3a). As was the case for BrdU labeling, the density of CD68 + cells did not further increase with larger MLL-tumors versus smaller MLL-tumors (Fig. 3a). Although there was a trend, the macrophage volume density was not significantly correlated with the volume density of BrdU (Rs = 0.355, n = 26, p = 0.075), suggesting that the increase in DNA synthesis was only weakly related to an increased macrophage activation/density in the liver.

In the lungs, macrophage density was significantly higher in animals with intra-prostatic MLL-tumors compared to tumor-free controls animals (Fig. 3b). In addition, the macrophage density increased with MLL-tumor size (Fig. 3b). Moreover, there was a correlation between BrdU volume density and macrophage volume density (Rs = 0.654, n = 27, p = 0.0002), suggesting that intra-prostatic MLL-tumors may induce macrophage accumulation/activation and a tissue repair response in the lungs.

**Figure 4:**
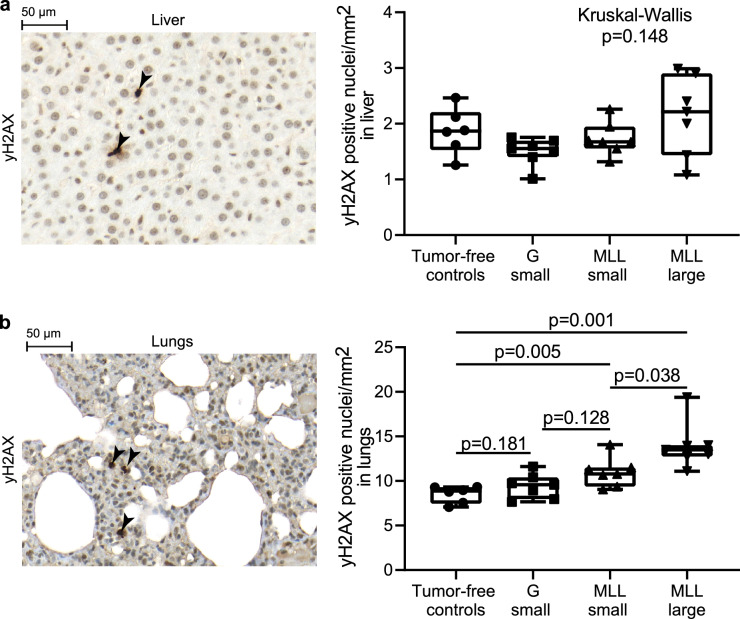
DNA-damage in liver and lungs in animals with intraprostatic tumors. Sections immune-stained for γH2AX (marker for DNA-damage) in liver (**a**) and lung tissue (**b**) in an animal with an intraprostatic MLL-tumor. Box plots showing the numbers of γH2AX + cells in liver (**a**) or lungs (**b**) of control rats, and in rats with intra-prostatic G or MLL-tumors. The number of γH2AX + cells was significantly increased in lungs by MLL-tumors compared to controls and in a tumor-size dependent way. The non-parametric Kruskal–Wallis H test was used for multiple comparison, if significant (p < 0.05), the non-parametric Mann–Whitney U test was used to compare two groups.

Tumors induce DNA damage in various distant organs and this effect is mediated by CCL2 secreted from host immune cells^28^. We therefore measured DNA-damage by immunostaining of γH2AX in liver and lungs (Fig. 4a,b). No significant difference in staining was found in the liver between animals with intra-prostatic tumors and controls, and the staining was not significantly changed with increased MLL-tumor size (Fig. 4a). In contrast, the number of γH2AX + cells in the lungs was significantly increased in animals with intra-prostatic MLL-tumors compared to controls (Fig. 4b). In addition, the number of γH2AX + cells in the lungs increased with increasing MLL tumor size (Fig. 4b). In lungs, BrdU and γH2AX volume densities were strongly correlated (Rs = 0.687, n = 27, p = 0.00008) whereas CD68 and γH2AX were less coupled but still significantly correlated (Rs = 0.397, n = 27, p = 0.04).

### Subcutaneous MLL-tumors increase BrdU-labeling in liver and lungs but not in the prostate

**Figure 5:**
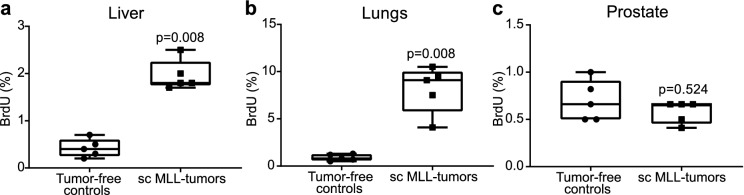
BrdU-labeling in liver, lungs and prostate in animals with subcutaneous tumors. Volume density (%) of BrdU + cells in the liver (**a**), lungs (**b**), and ventral prostate (**c**) in animals with subcutaneous (sc) MLL-tumors and in tumor-free controls. Sc MLL-tumors significantly increased BrdU-labeling in liver and lungs, while notably did not increase BrdU-labeling in the prostate (Mann–Whitney U test).

Increased BrdU-labeling in distant organs are likely caused by circulating factors induced by the tumor, while changes in the prostate could be due to both local paracrine signaling and/or systemic changes. We therefore examined BrdU-labeling in animals with large subcutaneous MLL-tumors (23.4 ± 2.9 g, mean ± SEM). Subcutaneous MLL-tumors resulted in a significant increase of BrdU-labeling in both liver and lungs compared to controls (Fig. 5a,b). However, large subcutaneous MLL-tumors had no effect on BrdU-labeling in the benign prostate (Fig. 5c). This suggests that increased BrdU-labeling in liver and lungs were caused by tumor-induced circulating factors while increased BrdU-labeling in the benign parts of the prostate was likely due to paracrine signals from the intra-prostatic tumors.

### Cytokine profiles in serum of animals with G or MLL tumors

Cytokine levels in serum from tumor-free rats and rats with similar sized subcutaneous G- or MLL-tumors (1.6 ± 0.5 g, and 1.9 ± 0.5 g, respectively, mean tumor weight ± SEM, p = 0.700, Mann–Whitney U test) were determined semi quantitatively using a Rat XL cytokine array.

**Figure 6:**
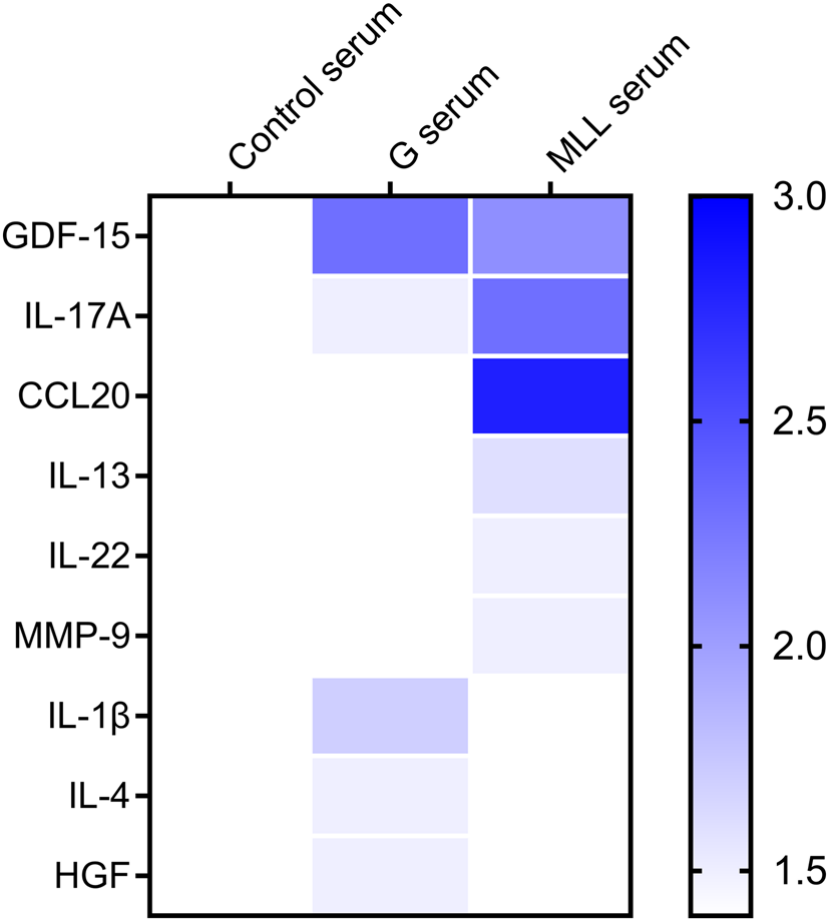
Cytokine protein profile in serum. Relative cytokine concentrations in serum from tumor-bearing animals (G serum and MLL serum) compared to serum from tumor-free controls (control serum, set as 1). The heatmap illustrates fold-change relative to levels in control-serum, white color indicates no changes compared to controls and blue color indicates higher levels (≥ 1.5-fold).

Serum from tumor-bearing rats had higher levels of 9 out of the 79 cytokines measured compared to serum from tumor-free controls (≥ 1.5-fold upregulation in any of the tumor groups) (Fig. 6, Table S2). Levels of 2 out of these 9 factors, GDF-15 and IL17A, were higher in serum from rats with both types of tumors compared to serum from controls (Fig. 6), suggesting that tumors increase these factors in blood regardless of their metastatic potential. Levels of 4 factors, CCL20, IL13, IL22 and MMP9, were higher only in serum from rats with MLL-tumors compared to controls, suggesting that some factors are more related to metastatic potential. Three factors, IL1β, IL4 and HGF, had higher levels in serum from rats with G tumors compared to the other two groups (Fig. 6).

## Discussion

Tumor-driven systemic processes promote primary tumor growth, pre-metastatic niche formation, metastatic colonization and cancer anorexia/cachexia^21,22,29,30^. If such systemic changes start already when tumors are small and if they can be used as biomarkers to improve early diagnosis of potentially lethal tumors is, however, largely unexplored. To start examining this possibility we used long-term BrdU-labeling as a sensitive method to detect early and discrete signs of tumor-induced tissue remodeling. We then compared how small organ-confined rat prostate tumors with different metastatic potential affected DNA synthesis in various remote tissues prior to detectable metastases. Notably, we cannot exclude the presence of single disseminated tumor cells in some organs, but if present their numbers are very low. Furthermore, the signs of tissue remodeling observed here occurred in other cell types than in tumor cells. Intra-prostatic tumors increased BrdU-labeling in multiple distant organs. The site, magnitude and nature of these changes were related both to tumor type (i.e. metastatic capacity) and to tumor size, and some occurred already when tumors were less than 3 mm in diameter.

Both low-metastatic G and high-metastatic MLL orthotopic tumors increased BrdU-labeling in the liver, suggesting that the liver may sense small prostate tumors, regardless of their aggressiveness. During inflammation in any organ, cytokines, like IL1, IL6 and TNFα, are secreted to the circulation. These cytokines activate Kupffer cells (stationary macrophages in the liver) and induce production of acute-phase proteins in hepatocytes^31^. As MLL-tumors express high levels of IL6, IL1β and TNFα^9^, changes in the liver could be related to a tumor-induced acute phase response. In line with this, IL1β was slightly increased in serum from rats with G tumors. Proteins secreted from the liver may in-turn affect tumor growth^21^ and possibly induce changes in other organs. In line with this, high levels of acute phase proteins in plasma, like C-reactive protein, are associated with disease aggressiveness in prostate cancer patients^32,33^. Further studies are needed to examine how different circulating factors affect remote organs, and their potential role as biomarkers.

Liver metastases are common in prostate cancer patients^34^, and in the rat tumor model, MLL-tumors sometimes metastasizes to the liver. This suggests that tumor-induced processes in the liver could also be involved in pre-metastatic niche formation. Exosomes derived from pancreatic cancer cells have been shown to induce ECM remodeling and recruitment of bone marrow-derived macrophages to the liver^35^. In line with this we here show that, intra-prostatic tumors gave rise to an increased density of CD68 + macrophages in the liver, presumably activated Kupffer cells and recruited macrophages, suggesting that circulating factors could be involved in tumor-induced liver alterations also in our model.

Interestingly, only intra-prostatic MLL-tumors were able to increase BrdU-labeling in the lungs, suggesting that this could be an organ that responds selectively to aggressive prostate tumors—and importantly in a tumor size-dependent way. Hypothetically, lungs could be a potential source for early biomarkers of aggressive prostate cancer. The first signs of microscopic lung metastases in our MLL-model, can be observed at day 18 (unpublished), and in men with prostate cancer, lungs are the second most common metastatic site after bone^34^. Early tumor-induced pre-metastatic changes, for example accumulation of inflammatory cells, could thus be present in the lungs of men with organ-confined potentially lethal prostate cancer, but this has to our knowledge not been examined. MLL-tumors and the surrounding tinted benign prostate tissue express high levels of CXCL12, S100A9, CCL2, IL1β, TNFα, G-CSF, and LOX^9^—all suggested to be involved in pre-metastatic niche formation in lungs and other tissues^21,22^. Some proteins, such as IL22, CCL20, and MMP9, all shown to be involved in tumor progression^36–38^ were exclusively higher in serum from rats with MLL-tumors.

Growth of intra-prostatic MLL-tumors was also associated with signs of DNA-damage and increased density of CD68 + macrophages (presumably stationary alveolar macrophages and recruited monocytes) in the lungs. Similarly, in other (non-prostate) tumor models in mice, Redon et al. showed that tumors induce DNA damage and a chronic inflammatory response in some distant tissues such as the lungs, and that CCL2 was essential for this process^28^. In this study, however, the levels of CCL2 were not higher in serum from tumor-bearing rats compared to controls. The reason for this discrepancy is unknown but other factors may be more important in our prostate cancer model. For example, CCL20, increased in serum from animals with MLL tumors, has been shown to be highly expressed in breast cancer models, and inhibition of CCL20 expression or depletion of the CCL20 receptor gene (*Ccr6*) reduce macrophage infiltration in xenografts^39,40^.

In breast cancer models, both myeloid-derived suppressor cells (MDSCs) recruited from the bone-marrow^41,42^ and resident alveolar macrophages^43^ form pre-metastatic niches by suppressing anti-tumor immune responses in the lungs and preparing the soil for later arriving tumor cells. Macrophages promote epithelial proliferation in the lungs following injury^44^. Increased BrdU-labeling in pneumocytes may be a sign of DNA-repair and/or of epithelial regeneration following tumor-induced tissue damage. Macrophages in the lungs could contribute to the damage and/or stimulate cell proliferation/repair in the epithelium in ways similar to that during wound healing.

Intra-prostatic MLL-tumors also resulted in increased BrdU-labeling in the bone marrow, although the magnitude of this was limited and apparently not tumor-size dependent. MLL-tumors do not form detectable bone metastases in rats, indicating that these changes are not necessarily pre-metastatic. Instead, primary tumor-derived signals may drive the expansion of hematopoietic stem cells and progenitor cells (HSPCs) in the bone marrow (assessed by increased BrdU incorporation) and their mobilization to the circulation^41^. These HSPCs differentiate into myeloid-derived suppressor cells at pre-metastatic sites and elevated levels of HSPCs in the circulation was associated with increased risk for metastasis in breast cancer patients^41^. Such a process may be selectively activated by highly aggressive tumors, like MLL, suggesting that tumor-promoting changes in the bone marrow could perhaps be used as early markers of potentially lethal disease. In line with this, alterations in the number of different leukocytes in blood, derived from the bone marrow, are already markers of disease aggressiveness in a variety of cancers, including the prostate^45,46^—further indicating that tumor-induced changes within the bone marrow and among circulating leukocytes may be of prognostic importance. Further pre-clinical and clinical studies of specific populations expanding in the bone marrow due to aggressive primary prostate cancer are needed. In patients, in sharp contrast to rodents, bone marrow is the most common metastatic site for prostate cancer. Changes in the bone marrow in men are therefore likely also coupled to pre-metastatic niche formation. This suggests that bone marrow may demonstrate primary tumor supporting changes, pre-metastatic changes, and later disseminated tumor cells—changes that could all be of prognostic importance.

MLL-tumors also affected skeletal muscle and brown adipose tissue in a tumor size-dependent way. Muscle and fat are involved in the cachexic process^47^. In this study, body weight was not significantly different in tumor-bearing rats vs. vehicle-injected controls, indicating that increased DNA synthesis in these organs could be an early sign of systemic effects of aggressive cancer. If these changes can be used to monitor disease aggressiveness remains to be explored. Interestingly, GDF15, a tumor derived factor mediating cancer cachexia^48^ is markedly over-expressed in MLL-tumors^9^ and GDF15 was higher in serum from rats with tumors.

Myeloid cells have been shown to expand in the spleen of tumor-bearing animals^49^, and shown to enhance breast tumor growth and metastasis^50^. In this study, BrdU-labeling in the spleen was increased due to surgery but no additional changes in BrdU-labeling were observed between vehicle-injected and tumor cell injected rats. Changes in the spleen was therefore difficult to evaluate in this model.

Organ confined rat prostate tumors induce changes in the rest of the tumor-bearing prostate lobe^6,8,9,51^. We now demonstrate that the magnitude of this response was related to the distance from the tumor. Moreover, subcutaneous growth of MLL-tumors did not influence prostate BrdU-labeling, suggesting that the signals *tinting* non-malignant cells in the prostate are mainly local and perhaps different from signals causing systemic effects. Increased BrdU-labeling in benign prostate epithelium could be mimicked by injecting MLL tumor-derived microvesicles into tumor-free prostate tissue, suggesting that some of the local effects could be mediated by extracellular vesicles^52^. In prostate cancer patients, increased epithelial levels of phosphorylated EGF-receptor and phosphorylated AKT in the benign tinted prostate epithelium were associated with raised epithelial cell proliferation, high disease aggressiveness and poor patient outcome^53,54^. In the majority of men with raised serum PSA, no tumor is found in subsequent prostate needle biopsies^55^. Epigenetic changes in the benign but tinted tissue sampled in such biopsies can actually be used to evaluate the risk that an aggressive tumor was missed^55^.

In conclusion, highly aggressive organ confined prostate cancers influence local and remote tissues already when the tumors are small. Hypothetically, such tumor-induced changes, when more specified, could perhaps be used as early diagnostic and prognostic biomarkers as well as novel therapeutic targets. Examining how different tissues are affected by small aggressive prostate cancers and how this can be differentiated from systemic changes induced by less aggressive tumors, could be done using a variety of methods. For example, by studying biomarkers in the circulation or by examining easily accessible cells/tissues such as circulating leukocytes, fat and skeletal muscle. It is also possible to use different imaging techniques. PET scanning has been used to visualize the accumulation of myeloid suppressor cells in pre-metastatic lung tissue in a breast cancer model^56^ and MR-spectroscopy can detect changes in liver metabolism in metastases-free animals carrying MLL-tumors^57^. How patient-derived serum and microvesicles affect the phenotype of various non-malignant cells (derived from different organs) in vitro could possibly also serve as a novel method to examine tumor aggressiveness, and is currently being examined.

## Methods

### Ethical statement

Animal experiments were approved by the Umeå Regional Animal Ethic Committee, Approval no. A42-15. All animal experiments were performed according to the approved protocols and in compliance with the ARRIVE guidelines.

### Implantation of rat prostate tumor cells

Immunocompetent adult Copenhagen rats (Charles River) were used in all experiments. G and MLL- rat prostate tumor cells were purchased from the European Collection of Cell Cultures (ECACC) and were grown in RPMI-1640/GlutaMax (Gibco) with 10% fetal bovine serum and 250 nM dexamethasone (Sigma Aldrich).

For intraprostatic implantation, animals were anesthetized (Ketamine; 75 mg/kg and Medetomidine; 0.5 mg/kg) and an incision was made in the lower abdomen to access the ventral prostate lobes. G cells (2 × 10^7^) or MLL cells (1 × 10^3^ or 2 × 10^3^) suspended in 10 µl of RPMI were carefully injected into one of the ventral prostate lobes (n = 7 for each group). The difference in number of cells injected is needed to reach the same tumor size after 10 days. Control animals received an intraprostatic injection of vehicle (RPMI, 10 µl) (n = 6). In addition, one group of controls were treatment-naïve (n = 6). All animals received daily intraperitoneal (ip) injections of BrdU (Sigma Aldrich, 50 mg/kg), with the last injection 1 h prior to sacrifice at day 10.

For subcutaneous implantation, MLL cells (2 × 10^5^ cells in 100 µl RPMI) or vehicle (100 µl RPMI) were injected subcutaneously (right flank, n = 5 in each group). Animals were sacrificed after 21 days, with daily BrdU injections for 5 consecutive days before sacrifice.

At sacrifice the animals were anesthetized and then euthanatized by removal of the heart. Prostates (tumor-containing and tumor-free), iliac lymph nodes, liver, spleen, lungs, tibia (bone marrow and skeletal muscle), and brown adipose tissue (peri-thymic) were removed and formalin-fixed for further analyzes.

To study circulating factors in serum, G tumor cells (1 × 10^8^ cells), MLL tumor cells (5 × 10^4^ cells), or vehicle (RPMI) were injected subcutaneously (n = 3 animals/group). To obtain approximately the same tumor sizes for both tumor types, animals with MLL tumor and controls were euthanized after 14 days and animals with G tumors after 33 days. Whole blood from anesthetized animals was collected from the heart into blood collection tubes (BD Vacutainer SST II Advance, BD, #368498), incubated at room temperature for 30 min, centrifuged at 1400×*g* for 15 min and serum was transferred to collection tubes and frozen at − 80 °C until use.

### Immunohistochemistry

All detection reagents and instruments were obtained from Ventana Medical Systems.

Tissues were single-stained for BrdU (BD Biosciences, # 347580, 1:200), CD68 (Bio-Rad, #MCA 341R, 1:200), or γH2AX (Novus Biologicals, #NB100-2280, 1:300) using the automated BenchMark ULTRA system with Cell Conditioning 1 (CC1) as antigen retrieval buffer and developed using the Ultraview Universal DAB Detection Kit. BrdU-staining required additional pretreatment with Protease 1 prior to antibody incubation.

The automated DISCOVERY ULTRA system was used for chromogenic double-staining of selected tissue sections (see Table 1 for details).

**Table 1:** Protocol parameters for DISCOVERY ULTRA chromogenic double-staining.

	CD3/BrdU	CD68/BrdU	CK14/BrdU	FVIII/BrdU	CK7/BrdU
HIER at 95 °C	CC1, 32'	CC1, 56'	CC1, 32'	CC1, 16'	CC1, 32'
Enzyme at 36 °C				Protease 2, 4'	
Antibody at 36 °C	CD3 (Abcam #ab16669) 1:50, 32'	CD68 (Bio-Rad, #MCA 341R), 1:200, 32'	CK14 (Biosite, #PRB-155P-100), 1:200, 32'	FVIII (DAKO, #A0082), 1:1000, 24'	CK7 (Atlas Antibodies, #HPA007272), 1:200, 32'
Multimer	UltraMap anti-Rb AP, 16'	UltraMap anti-Ms AP, 16'	UltraMap anti-Rb AP, 16'	UltraMap anti-Rb AP, 16'	UltraMap anti-Rb AP, 16'
Chromogen	DISCOVERY RUO Yellow, 44'	DISCOVERY RUO Yellow, 44'	DISCOVERY RUO Yellow, 44'	DISCOVERY RUO Yellow, 44'	DISCOVERY RUO Yellow, 44'
Antibody Denaturation at 100 °C	CC2, 8'	CC2, 8'	CC2, 8'	CC2, 8'	CC2, 8'
Enzyme 36 °C	Protease 2, 4'	Protease 2, 4'	Protease 2, 4'		Protease 2, 4'
Antibody at 36 °C	BrdU; 1:50, 32'	BrdU; 1:50, 32'	BrdU; 1:50, 32'	BrdU; 1:50, 32'	BrdU; 1:50, 32'
Multimer	OmniMap anti-Ms HRP, 16'	OmniMap anti-Ms HRP, 16'	OmniMap anti-Ms HRP, 16'	OmniMap anti-Ms HRP, 16'	OmniMap anti-Ms HRP, 16'
Chromogen	DISCOVERY RUO Purple, 32'	DISCOVERY RUO Purple, 32'	DISCOVERY RUO Purple, 32'	DISCOVERY RUO Purple, 32'	DISCOVERY RUO Purple, 32'
Hematoxylin II	4'	4'	4'	4'	4'
Bluing Reagent	4'	4'	4'	4'	4'

Endogenous peroxidase was quenched with DISCOVERY Inhibitor. All antibodies were diluted in DISCOVERY ab Dilution.

The volume density of BrdU, CD68, CD68 + /BrdU +, or CK7 + /BrdU + labeled cells were measured using a 121-point square lattice mounted in the eye-piece of a microscope. A selection of BrdU stained samples were also quantified using the using the QuPath software version 0.2.0^58^ giving similar results (Fig. S2). γH2AX were quantified as positive cells/mm^2^ using the Panoramic Viewer software version 1.15.2 (3DHistech). γH2AX was counted as positive when nuclear foci were pronounced. Tumor size was also calculated by measuring the volume density of tumor and multiplying it with prostate weight as earlier described^6^ and as this was highly correlated to maximal tumor cross sectional area (R_s_ = 0.839, n = 20, p = 0.000004) we used cross- sectional area as estimate of tumor size.

### Rat cytokine array

Cytokines were examined in serum (pooled from 3 animals/group) from animals with subcutaneous G or MLL tumors and tumor-free animals using the Proteome Profiler Rat XL Cytokine Array Kit (R&D systems) according to the manufacturer’s instructions. Briefly, 200 µl of each serum was added to the membranes and incubated overnight at 5 °C on a rocking platform shaker. Detection antibodies were thereafter added to each membrane. After one hour of incubation at room temperature, Streptavidin-HRP was added to detect cytokines and then visualized using a Chemi Reagent Mix. Visualization was performed with the ChemiDoc Imaging System (BioRad) and spot pixel densities were analyzed using the ImageLab software 5.2.1 (BioRad). After subtraction of background signal, the relative change in analyte levels between samples were determined using tumor-free serum as a reference group.

### Statistical analysis

Statistical analysis was performed with the GraphPad Prism 7 Software (GraphPad Software Inc.) or SPSS Statistics 26 (SPSS Inc.). The non-parametric Kruskal–Wallis H test was used for multiple comparisons. The non-parametric Mann–Whitney U test was used when comparing two groups. The Spearman’s rho test was used for correlations and the Wilcoxon test was used for paired sample analysis.

## Supplementary Information

Supplementary Information.

## Data Availability

Data generated or analyzed during this study are included in this published article and its supplementary information files or available from the corresponding author on reasonable request.
