# Supplementary material for: Rat prostate tumors induce DNA synthesis in remote organs
Source: Sci Rep. 2022 May 12;12:7908. doi: 10.1038/s41598-022-12131-6 (PMC9098422; doi:10.1038/s41598-022-12131-6)
Supplement: Supplementary file 1 — Supplementary Information. [file 41598_2022_12131_MOESM1_ESM.pdf]

Supplementary information:

Rat prostate tumors induce DNA synthesis in remote organs

Sofia Halin Bergström[1]*, Marie Lundholm[1], Annika Nordstrand[1], and Anders Bergh[1]

[1]Department of Medical Biosciences, Pathology, Umeå University, Umeå, Sweden

Figure S1

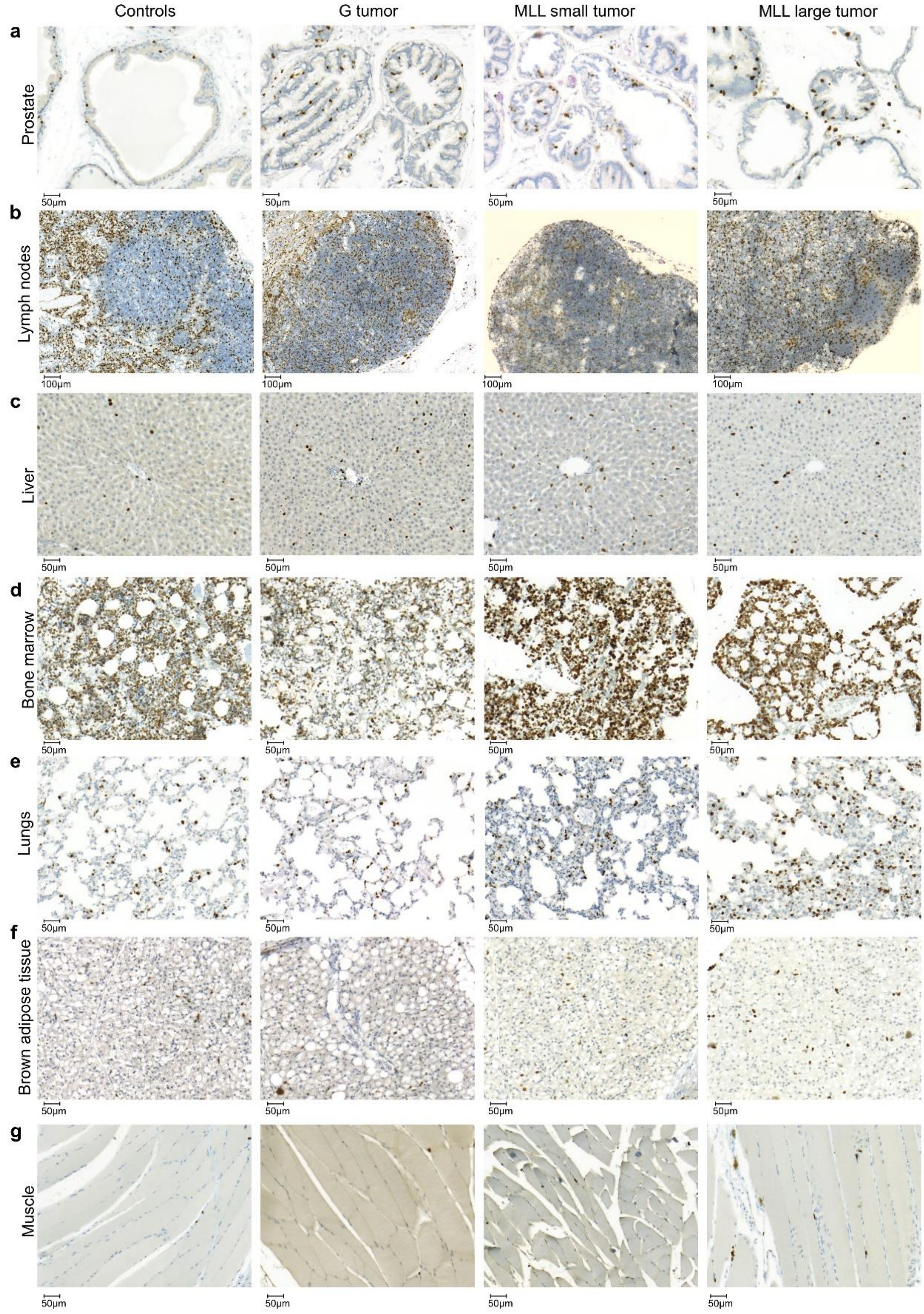

Figure S1. **BrdU staining in various organs with intraprostatic tumors.** BrdU-stained sections from **a**) prostate, **b**) lymph nodes, **c**) liver, **d**) bone marrow, **e**) lungs, **f**) brown adipose tissue, and **g**) muscle in; a control rat, a rat with an intraprostatic G-tumor (G tumor), a rat with an intraprostatic MLL-tumor of similar size as the G-tumor (MLL small tumor), and a rat with a larger intraprostatic MLL-tumor (MLL large tumor). Intraprostatic tumors increased BrdU-labeling in various organs in relation to tumor type and size. For quantification of BrdU see figure 1.

Figure S2

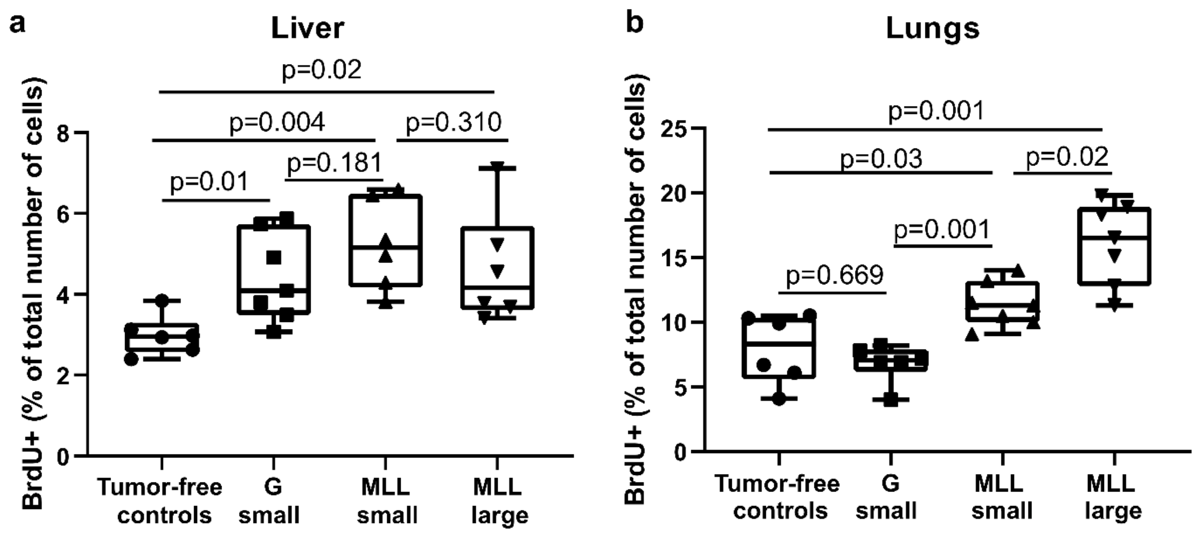

Figure S2. BrdU-labeling in liver and lungs in animals with intraprostatic tumors. The numbers of BrdU positive cells in relation to the total number of cells (%) were analyzed in **a**) liver and **b**) lungs of tumor-free rats and in rats with intraprostatic tumors using the Qupath software (showed as box plots). An average of approximately 75 000 cells was analyzed in each liver and 140 000 cells for each lung. The results are comparable to results of BrdU volume density showed in figure 1.

**Table S1: BrdU quantification in various tissues of tumor-free control rats**			
	BrdU-labeling (%, mean +/- SEM)		
	Rats with surgery and intraprostatic vehicle injection	Treatment-naïve rats	P-value
Prostate	0.7 +/-0.1	0.2 +/- 0.06	0.007
Lymph nodes	9.6 +/- 1.4	6.2 +/- 0.9	0.121
Liver	0.3 +/- 0.05	0.3 +/- 0.03	0.677
Lungs	2.0 +/- 0.2	2.0 +/- 0.2	0.744
Bone marrow	61.0 +/- 1.1	59.3 +/- 2.3	0.630
Brown adipose tissue	0.25 +/- 0.02	0.30 +/- 0.07	0.414
Muscle	1.0 +/- 0.18	1.2 +/- 0.05	0.375
Spleen	15.3 +/- 0.9	12.5 +/- 0.4	0.015

Table S2: Cytokine array data						
	Adjusted volume intensities (Volume intensity- background)			Fold-change (FC) compared to control serum		
	**Control serum**	**G serum**	**MLL serum**	**FC control**	**FC G**	**FC MLL**
Adiponectin/Acrp30	1943121	2054285	1762582	1.0	1.1	0.9
CCL2/JE/MCP-1	744557	858531	780045	1.0	1.2	1.0
CCL3/CCL4/MIP-1α/β	196748	216867	222697	1.0	1.1	1.1
CCL5/RANTES	2265947	2218994	2326722	1.0	1.0	1.0
CCL11/Eotaxin	1619327	1654416	1660405	1.0	1.0	1.0
CCL17/TARC	221343	213442	224180	1.0	1.0	1.0
CCL20/MIP-3α	172523	212909	480104	1.0	1.2	2.8
CCL21/6Ckine	568921	610613	682210	1.0	1.1	1.2
CCL22/MDC	647061	654002	756048	1.0	1.0	1.2
Clusterin	587446	587146	556320	1.0	1.0	0.9
CNTF	177864	187669	194546	1.0	1.1	1.1
CX3CL1/Fractalkine	284296	300844	269916	1.0	1.1	0.9
CXCL2/GROβ/MIP-2/CINC-3	166472	163012	161881	1.0	1.0	1.0
CXCL7/Thymus Chemokine-1	1066788	952271	997374	1.0	0.9	0.9
Cyr61/CCN1	365450	391115	384779	1.0	1.1	1.1
Cystatin C	1120646	1162627	1110189	1.0	1.0	1.0
DPPIV/CD26	1180438	1210115	1098307	1.0	1.0	0.9
EGF	139890	145546	154038	1.0	1.0	1.1
EG-VEGF/PK1	144485	119085	143445	1.0	0.8	1.0
Endostatin	1349560	1475050	1297877	1.0	1.1	1.0
Fetuin A/AHSG	1304834	1255289	1125569	1.0	1.0	0.9
FGF acidic	183673	201935	190629	1.0	1.1	1.0
FGF-7/KGF	94948	91978	105626	1.0	1.0	1.1
FGF-21	301594	309840	322549	1.0	1.0	1.1
Fibulin	872088	892656	849451	1.0	1.0	1.0
Flt-3 Ligand	717222	738010	742475	1.0	1.0	1.0
Galectin-1	245045	239334	232745	1.0	1.0	0.9
Galectin-3	620684	626796	657057	1.0	1.0	1.1
G-CSF	141041	136251	147040	1.0	1.0	1.0
GDF-15	87627	203604	185785	1.0	2.3	2.1
GM-CSF	125075	125844	151242	1.0	1.0	1.2
Hepassocin	706191	805507	707935	1.0	1.1	1.0
HGF	298726	450358	388857	1.0	1.5	1.3
ICAM-1/CD54	734565	731255	656623	1.0	1.0	0.9
IFN-γ	172765	246580	203171	1.0	1.4	1.2
IGF-I	1625307	1605988	1402514	1.0	1.0	0.9
IGFBP-2	1086171	1192012	1025619	1.0	1.1	0.9
IGFBP-3	2116446	2201991	1891470	1.0	1.0	0.9
IGFBP-5	1079082	1294245	928021	1.0	1.2	0.9
IGFBP-6	1421883	1490296	1200345	1.0	1.0	0.8
IL-1α/IL-1F1	152853	206802	169307	1.0	1.4	1.1
IL-1β/IL-1F2	103520	174474	142360	1.0	1.7	1.4
IL-1ra/IL-1F3	132510	141022	142104	1.0	1.1	1.1
IL-2	181432	190360	176705	1.0	1.0	1.0
IL-3	211299	289708	234836	1.0	1.4	1.1
IL-4	125051	186119	167229	1.0	1.5	1.3
IL-6	136381	186598	157353	1.0	1.4	1.2
IL-13	116414	148848	182090	1.0	1.3	1.6
IL-17A	17568	26414	39634	1.0	1.5	2.3
IL-22	68438	80190	101971	1.0	1.2	1.5
Jagged	599258	691782	616912	1.0	1.2	1.0
LIF	205386	244417	225448	1.0	1.2	1.1
Lipocalin-2/NGAL	1150887	1214906	1050470	1.0	1.1	0.9
LIX	1596769	1894825	1496086	1.0	1.2	0.9
MAG/Siglec-4a	185996	189801	206110	1.0	1.0	1.1
MMP-2	1379676	1288744	1147446	1.0	0.9	0.8
MMP-3	449707	540297	416173	1.0	1.2	0.9
MMP-9	425846	423305	619617	1.0	1.0	1.5
Neprilysin/CD10	213771	246471	204655	1.0	1.2	1.0
NOV/CCN3	747090	814720	748537	1.0	1.1	1.0
NT-3	167834	201388	169386	1.0	1.2	1.0
NT-4	110302	127295	122147	1.0	1.2	1.1
Osteopontin (OPN)	922461	1002333	898140	1.0	1.1	1.0
Osteoprotegerin/TNFRSF11B	422213	465699	380139	1.0	1.1	0.9
PDGF-BB	274773	388304	297102	1.0	1.4	1.1
Pref-1/DLK1/FA1	405267	448835	372892	1.0	1.1	0.9
Prolactin	719477	733635	685922	1.0	1.0	1.0
RAGE	549497	672328	689264	1.0	1.2	1.3
RBP4	1002695	1027993	920281	1.0	1.0	0.9
Resistin	844779	644220	623995	1.0	0.8	0.7
RGM-A	707367	742568	616008	1.0	1.0	0.9
SCF	192398	193597	184594	1.0	1.0	1.0
Serpin E1/PAI-1	276756	364980	310047	1.0	1.3	1.1
TIM-1/KIM-1/HAVCR	251240	289655	237216	1.0	1.2	0.9
TNF-α	147021	176901	161604	1.0	1.2	1.1
TWEAK/TNFSF12	147489	167343	135802	1.0	1.1	0.9
VCAM-1/CD106	959737	1077251	926210	1.0	1.1	1.0
VEGF	156367	181477	157423	1.0	1.2	1.0
WISP-1/CCN4	570936	491835	458729	1.0	0.9	0.8
